# Wet-Etched Microlens Array for 200 nm Spatial Isolation of Epitaxial Single QDs and 80 nm Broadband Enhancement of Their Quantum Light Extraction

**DOI:** 10.3390/nano11051136

**Published:** 2021-04-27

**Authors:** Shulun Li, Xiangjun Shang, Yao Chen, Xiangbin Su, Huiming Hao, Hanqing Liu, Yu Zhang, Haiqiao Ni, Zhichuan Niu

**Affiliations:** 1 State Key Laboratory for Superlattice and Microstructures, Institute of Semiconductors, Chinese Academy of Sciences, Beijing 100083, China; lishulun@semi.ac.cn (S.L.); chenyao@semi.ac.cn (Y.C.); suxb@semi.ac.cn (X.S.); hmhao@semi.ac.cn (H.H.); hqliu@semi.ac.cn (H.L.); zhangyu@semi.ac.cn (Y.Z.); zcniu@semi.ac.cn (Z.N.); 2 Center of Materials Science and Optoelectronics Engineering, University of Chinese Academy of Sciences, Beijing 100049, China; 3 Beijing Academy of Quantum Information Sciences, Beijing 100193, China; 4 Institute of Photonics and Photonic Technology, Northwest University, Xi’an 710127, China

**Keywords:** microlens array, single photon, quantum dot, extraction efficiency

## Abstract

Uniform arrays of three shapes (gauss, hat, and peak) of GaAs microlenses (MLs) by wet-etching are demonstrated, ∼200 nm spatial isolation of epitaxial single QDs embedded (λ: 890–990 nm) and broadband (Δλ∼80 nm) enhancement of their quantum light extraction are obtained, which is also suitable for telecom-band epitaxial QDs. Combined with the bottom distributed Bragg reflector, the hat-shaped ML forms a cavity and achieves the best enhancement: extraction efficiency of 26%, Purcell factor of 2 and single-photon count rate of $7\times 10^{6}$ counts per second at the first lens; while the gauss-shaped ML shows a broader band (e.g., longer λ) enhancement. In the MLs, single QDs with featured exciton emissions are observed, whose time correlations prove single-photon emission with multi-photon probability $g^{\left(2\right)}\left(0\right)=0.02$; some QDs show both biexciton XX and exciton *X* emissions and exhibit a perfect cascade feature. This work could pave a step towards a scalable array of QD single-photon sources and the application of QD photon-pair emission for entanglement experiments.

## 1. Introduction

Quantum light source is an essential device in quantum communication systems and other applications in quantum technology [1,2,3] such as quantum-enhanced sensing [4] or imaging [5]. Semiconductor quantum dots (QDs) is a promising candidate for realization of on-demand quantum light source with high photon extraction efficiency, strong suppression of multi-photons and high indistinguishability of the emitted photons [6]. However, unlike colloidal QDs in solution which can be diluted easily, low-density epitaxial QDs needs a well control of the deposition amount during epitaxy, and their density is usually correlated with the QD size. Epitaxial low-density (∼1–2 μm${}^{-2}$) InAs/GaAs QDs usually show exciton emission at the wavelength (λ) of 900–930 nm, quite smaller than ensembled QDs with λ at ∼1300 nm. A well spatial isolation by etching might create longer-λ single QD regions for quantum application. Moreover, to overcome a large total reflection (98%) at the semiconductor/air interface and improve the light extraction efficiency (η) of a QD in it, the common solution is coupling the QD with dielectric antenna [7], solid-immersed lens [8], micro-cavity such as micropillars [9], microdisk [10], and photonic-crystal cavity [11], or waveguide [12]. For photon-pair entanglement application, both biexciton XX and exciton *X* emissions separated in λ of 2–3 nm must be enhanced that cannot be achieved by a high-Q cavity with a narrow-linewidth cavity mode; hence, several broadband-enhanced photonic structures such as circular Bragg grating cavity [13,14,15,16], metallic nanorings [17], photonic trumpet [18], microlens (ML) [19,20,21] and solid-immersed lens (SIL) [22] have been developed.

In this work, we fabricate GaAs MLs in three shapes (peak, gauss, and hat) by wet-etching method. Compared to the previously reported MLs fabricated by electron beam lithography) [19,20], the wet-etched MLs in this work are excellent on surface roughness (∼0.5 nm), convenient and low-cost for scalable uniform fabrication, small size for ∼200 nm spatial isolation of single QDs embedded (λ: 890–990 nm, suitable for telecom-band QDs too), broadband (Δλ∼ 80 nm) enhancement of their quantum light extraction, and reveal a Purcell enhancement effect. The η of single QD in the ML reaches 26% at highest (15 times enhancement with respect to the flat sample, η=1.7%); combining with the bottom distributed Bragg reflector (DBR), the hat-shaped ML forms a cavity and achieves a Purcell enhancement factor of ∼2 (with the QD at the center) and a total single-photon extraction rate at the first lens of $7\times 10^{6}$ counts per second (cps). Furthermore, the wet-etched MLs are compatible for the deterministic fabrication on proper QDs by a convenient low-temperature in situ photolithography technique [23,24,25] and suitable for the opto-electronic device applications such as light emitting diodes [26,27] and photon detectors [28].

## 2. Materials and Methods

The sample is grown by a solid-source molecular beam epitaxy system on semi-insulating (100) GaAs substrate. The epitaxial structure consists of a 500 nm-thick GaAs buffer layer, 15.5 pairs of lattice-matched Al${}_{0.9}$Ga${}_{0.1}$As/GaAs DBRs with a designed λ of 920 nm, and a 700 nm-thick top GaAs layer for ML fabrication. Active InAs QDs are inserted in the top GaAs layer at a distance of 396 nm (∼3λ/2n) from the top AlGaAs DBR layer. To obtain low-density (∼1–2 μm${}^{-2}$) QDs, a sacrificed QD layer is first deposited to monitor the critical indium coverage ($\theta_{c}$) for 2D to 3D transition with reflection high-energy electron diffraction [29] and then the formal QD layer is grown with a gradient indium flux (growth rate: 0.005 monolayer/s) and a subcritical deposition amount (1.80 monolayers) [30,31].

**Figure 1:**
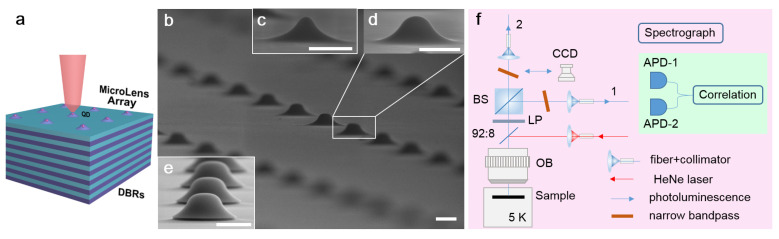
(**a**) Schematic of ML-DBR hybrid structure; (**b**–**e**) Scanning electron microscope (SEM) images of MLs in different shapes: (**b**) bird-view of ML array; (**c**) peak; (**d**) gauss; and (**e**) hat. Scale bars: 1 μm. (**f**) Confocal and HBT setups for μPL spectroscopy and photon correlation measurement.

**Figure 2:**
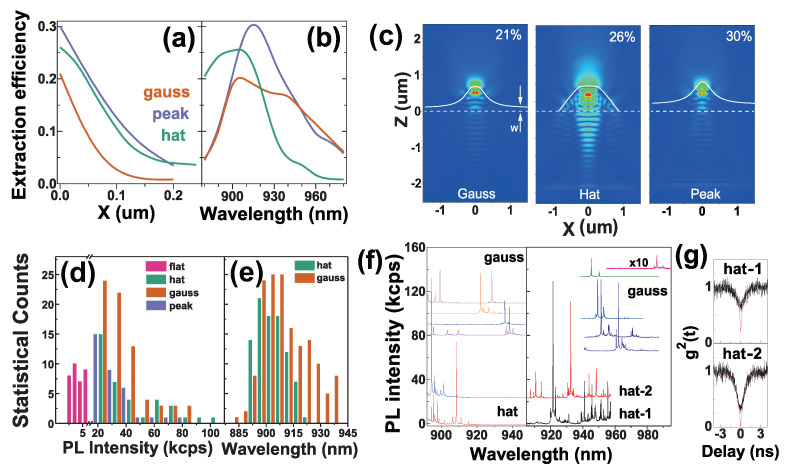
Simulation of light extraction efficiency η of a QD (at Z = 0.396 μm) in a ML, as a function of QD horizontal position X (**a**) and wavelength (**b**); (**c**) typical YZ-cut profile electric field distribution in a ML (η is given), W: the ML vertical position from the top AlGaAs DBR, the optimal W is 0.116 μm (gauss), 0.012 μm (hat) and 0.080 μm (peak); statistics on QD exciton emission peak intensity (**d**) and wavelength (**e**) in a total of 176 spectral lines, 34 in the flat sample and 142 in different shaped MLs; (**f**) typical μPL spectra of QDs in the hat- and gauss-shaped ML, offset vertically for clarity: MLs are fabricated in the dilute QD region (left) or the multi-QD region (right); (**g**) auto-correlations of the dominant peaks in the hat-shaped MLs hat-1 and hat-2, with radiative lifetimes extracted from their photon autocorrelation, 0.3 ns for hat-1 and 0.6 ns for hat-2, red: deconvolution fitting, blue: convolution fitting.

GaAs microlens arrays are fabricated as follows: (1) negative-glue photolithography is performed to prepare circular holes array (diameter: 3 μm, period: 10 μm) and a 150 nm-thick ${\mathrm{SiO}}_{2}$ Ion Beam sputtering and then the liftoff process is used to fabricate ${\mathrm{SiO}}_{2}$ hard mask array; (2) a buffered oxide etching (BOE) process is implemented to reduce the ${\mathrm{SiO}}_{2}$ mask diameter to 1.5–2.0 μm; (3) isotropic wet-etching of GaAs bulk at the edge of the mask is performed by $H_{2}{\mathrm{SO}}_{4}$$:H_{2}O$$:H_{2}O_{2}$ solution [21] and a hillock-like ML is formed under the ${\mathrm{SiO}}_{2}$ mask as the etching time increases. The $H_{2}O_{2}$ concentration is key for the etching rate and the final ML height (*H*) and base distance from the top AlGaAs DBR layer (*W*). By precisely controlling the mask diameter, the etching time and the $H_{2}O_{2}$ concentration, uniform arrays of MLs in three shapes with smooth surface are realized, as shown in Figure 1: (c) peak; (d) gauss; (e) hat-shaped MLs array and the detailed shape parameters is given by Table 1. The ML waist (FWHM) is 0.59 μm (peak), 0.61 μm (gauss) and 1.04 μm (hat) with a spatial isolation of single QDs of 200 nm at most as shown in Figure 2a.

To evaluate the enhancement of the light extraction from a QD embedded in the ML, we perform micro-photoluminescence (μPL) spectroscopy through the experimental setup shown in Figure 1f. The sample is cooled to the temperature (T) ∼5 K in a helium-flow cryostat and excited by a continuous-wave HeNe laser (λ = 632.8 nm) under saturated conditions. The confocal microscope setup uses an objective (OB, numerical aperture = 0.7) to focus the laser spot in a diameter of 2 μm (assisted by CCD imaging) to excite the QDs and collect their photoluminescence (blue) after a 92% transmission/8% reflection (92:8) beamsplitter and a longpass (LP) with collimators and multi-mode fibers. The spectrograph is performed with a monochromator equipped with a liquid-nitrogen-cooled Si CCD detector. To characterize the quantum light emission (e.g., single-photon count rate, purity and cascade behavior), photon correlation is measured by an integrated Hanbury Brown-Twiss (HBT) setup: a 50:50 beamsplitter (BS) divides the photoluminescence equally; a narrow-linewidth interference bandpass (Δλ = 0.4 nm) is inserted in each beam to filter a spectral line before fiber collection [32]; each fiber output connects a Si avalanched single-photon counter (APD-1, 2, time resolution: 350 ps) for detection; time correlation is analyzed by a time-to-digital converter with multi-channel buffer; the whole instrument response function (IRF) time is T${}_{IRF}$∼ 600 ps that is used for the deconvolution of the measured correlation data [33] to reflect the real one. Concerning the Si APD single-photon counter quantum efficiency (33% at λ∼ 900 nm) and the confocal setup efficiency (∼40%, i.e., a product of the collimator and multi-mode fiber coupling efficiency 67%, the filter-set transmission efficiency 70% and the objective collection efficiency 85%), the whole collection efficiency at the two APDs is 13.2%, higher than that of the traditional setup (2–3%) with a grating as a filter which has multi-order diffraction. The whole collection efficiency is used to estimate the net single-photon count rate at the first lens. Numerical simulations are performed through the finite time difference domain (FDTD) method to discuss the microlens enhancement of QD light extraction. In the simulation model, the ML surface topography is extracted from the SEM images in Figure 1 and the QD is simplified as a continuous-wave dipole emitter; the light extraction efficiency η is estimated through a time monitor above the ML with a collection numerical aperture of 0.7, divided by the light output in all space.

## 3. Results and Discussion

Figure 2 presents the simulation of the light extraction efficiency η as a function of QD horizontal position X concerning the ML center and QD emission wavelength, the typical μPL spectra of QDs, and the statistics on QD exciton emission spectral lines. Figure 2d is statistics on PL peak intensity. The intensity of QDs in the flat sample varies from 2 kcps to 5.5 kcps with the average $I_{flat}$ of 4 kcps, while the intensity of QDs in the MLs varies from 20 kcps to 110 kcps at maximum (hat-shaped). Its statistical distribution is a good reflection of the QD X-position variation from the ML center as the simulation in Figure 2a reflects: the maximal intensity corresponds to a QD in the ML center that has very few statistical counts, while the minimal intensity (∼20 kcps) with many statistical counts corresponds to a QD away from the ML center that is popular. For the optimal QD position (x = 0, z = 0.396 μm), Figure 2c presents the simulated typical electric field distribution in a ML, with the η of 21%, 26% and 30% in gauss-, hat- and peak-shaped MLs, respectively. The enhancement factor *F* is defined as the ratio of the maximal PL intensity in a ML (i.e., QD at the ML center) and the average PL intensity in the flat samples $F=I_{ML}/I_{flat}$. The hat-shaped MLs achieve a greater F=110/4=28 at maximum while the gauss-shaped ones achieve an F=55/4=14 at maximum (corresponding to the optimal η=14×1.7%=24%). Figure 2f presents the typical μPL spectra of QDs in the hat- and gauss-shaped MLs fabricated in the dilute QD region (left) where single QD is observed in a ML and in the multi-QD region (right) where multi-QDs are observed, especially in a hat-shaped ML. In both regions, the gauss-shaped MLs show single QDs with longer λ of 930–970 nm and even reach 990 nm, reflecting well spatial isolation of single epitaxial QDs. As the simulation results in Figure 2a shows, the QD X-variation from the ML center plays a significant role on the η, therefore, the MLs offer ∼200 nm spatial isolate of single QDs (i.e., FWHM ∼200 nm of the η as a function of the QD X-position) as the clean μPL spectra of single QDs in Figure 2f reflects. The spatial isolation of these MLs can also be used in telecom-band epitaxial QDs [31]. As Figure 2f shows, the hat-shaped MLs mainly enhance QD emission at shorter λ with a greater *F* = 28, while the gauss-shaped MLs enhance QD emission at longer λ with *F* = 14. The ultra-high *F* in the hat-shaped ML is due to a cavity Purcell enhancement, as the simulated light field distribution in Figure 2c indicates, the hat-shaped ML combined with the bottom DBR shows a normal-directional light field distribution and extraction like a DBR cavity and the internal light field is focused at the ML center (i.e., QD location) for a high Purcell enhancement with Purcell factor of ∼2 that leads to the ultra-high *F* (i.e., 14 × 2 = 28), in contrast with the divergent light field extraction from the gauss- and peak-shaped MLs. The Purcell enhancement effect is also reflected from the radiative lifetime extracted from photon autocorrelation, which is 0.3 ns in a hat-shaped ML (hat-1) with a maximal PL intensity (130 kcps at peak) while 0.6 ns in another hat-shaped ML (hat-2) with a lower intensity (80 kcps at peak), as shown in Figure 2f,g. Figure 2e is the statistics on QD emission wavelength, in agreement with the simulation results of the wavelength dependence of the η in Figure 2b. A broadband (Δλ∼80 nm) enhancement is reflected; the hat-shaped MLs mainly enhance QD light extraction in a shorter λ: 900–930 nm while the gauss-shaped MLs enhance it in a wider range, 890–970 nm, also confirmed by the typical μPL spectra in Figure 2f. The net single-photon count rate of the QD-in-MLs is estimated by the total APD photon count rate divided by the whole collection efficiency in the HBT setup (13.2%). For the hat-shaped ML (hat-1 in Figure 2f with PL peak intensity beyond 130 kcps), its total photon count rate at the two APD detectors is $0.9\times 10^{6}$ cps and the net single-photon count rate is $7\times 10^{6}$ cps at the first objective lens, with extraction efficiency η of 26% and a Purcell factor ∼2. The extraction efficiency can be further improved if the bottom DBR is replaced with a gold mirror via substrate removing processing [19].

**Figure 3:**
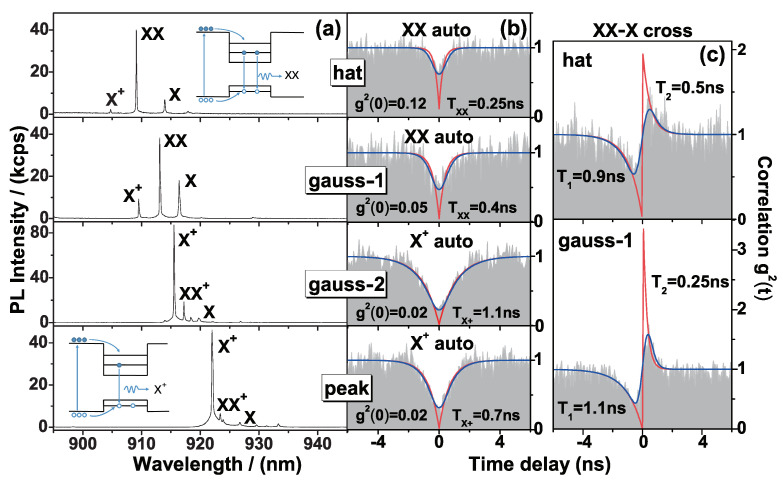
Typical μPL spectra (**a**) and secondorder autocorrelation function $g^{\left(2\right)}\left(\tau \right)$ (**b**) of QDs in the three types of MLs, red: deconvolution fitting, blue: convolution fitting. (**c**) XX–*X* cross-correlation function $g^{\left(2\right)}\left(\tau \right)$ of QDs in the gauss-1 and hat MLs. Inset: theoretical schemes of QD biexciton (XX) and charged exciton ($X^{+}$) transition.

To confirm single-photon emission from the QDs in the MLs, we select representative µPL spectra of each type of MLs to measure second-order photon correlation function $g^{\left(2\right)}\left(\tau \right)$ via the HBT setup. The results are shown in Figure 3. Exciton spectral peaks are attributed relying on their excitation power dependences and spectral features that nearly keep the same for different QDs [34]. Under saturated excitation, biexciton XX emission is dominant for QDs in the hat- (hat) and gauss-shaped MLs (gauss-1), their different radiative lifetimes from photon autocorrelation, $\tau_{XX}$ = 0.25 ns and 0.4 ns, respectively, smaller than the typical XX radiative lifetime 0.5 ns, together with the ultra-high maximal PL intensity in the hat-shaped MLs in Figure 2f, reflect a cavity Purcell enhancement factor of ∼2. The cavity effect in the hat-shaped MLs also induces a strong XX repopulation and a high multi-photon probability $g^{\left(2\right)}\left(0\right)$, 0.12 in the hat ML in Figure 3 and 0.2 in the hat-1 ML in Figure 2 (radiative lifetime τ = 0.3 ns) after deconvolution. The charged exciton $X^{+}$ emission dominates in a peak-shaped ML (peak) and another gauss-shaped ML (gauss-2) with radiative lifetime of 0.7 ns and 1.1 ns in the same level with no cavity effect and exhibits the best single-photon purity $g^{\left(2\right)}\left(0\right)$ = 0.02 with no background subtraction correction. For the two gauss-shaped MLs, consistent with a normal situation [35], the XX radiative lifetime is nearly one-half of the *X* (or $X^{+}$) radiative lifetime and the $X^{+}$ emission is brighter than *X*, since the electron-hole recombination rate of the biexciton XX with two pairs of electrons and holes is twice as much as that of a single exciton (*X* or $X^{+}$) with only one pair of electrons and holes, as the transition schemes in the inserted figures exhibited.

Figure 3c presents the typical cross-correlations of the XX-*X* cascade emission in QDs in the hat- and gauss-shaped MLs. Concerning the hat-shaped ML with intense XX emission but weak *X* emission due to a strong XX repopulation before *X* emission, the QD in gauss-1 ML shows comparable XX and *X* emission intensity which exhibit a good cascade feature, with the bunching $g^{\left(2\right)}\left(0\right)$ peak as high as 3.3 and the antibunching $g^{\left(2\right)}\left(0\right)$ dip as low as 0 after deconvolution, quite suitable for photon-pair entanglement application [32]. The asymmetric cross-correlation of the XX-*X* cascade in Figure 3c can be theoretical explained as follows: in the positive time delay (i.e., *X* is subsequent to XX), the clear bunching peak reflects a cascade *X* exciton emission after the biexciton XX emission that transits to the exciton state |X〉; in the negative time delay (i.e., *X* is ahead of XX), the antibunching dip is due to the repopulation of the biexciton state |XX〉 from the ground state |G〉 (i.e., vacuum of electron and hole in QD) after *X* exciton emission. For the exciton state |X〉, apart from a direct electron-hole recombination to emit an *X* photon, there is also a considerable probability of repopulation of the biexciton state |XX〉 via carrier capture and in this case the biexciton XX emission is dominant among the multi-exciton emissions, as the case in Figure 3a, the hat and gauss-1 shaped MLs, reflect. The characteristic time of the bunching peak $T_{2}$ (reflecting cascade emission rate) is only 0.25 ns, smaller than that in the hat-shaped ML ($\tau_{X}$ = 0.5 ns), while the characteristic time of the antibunching dip $T_{1}$ (i.e., carrier population rate for XX) is 1.1 ns, larger than the $T_{1}$ in hat-shaped ML, 0.9 ns, reflecting a well XX-*X* cascade emission instead of a dominant XX repopulation.

## 4. Conclusions

In conclusion, we demonstrate uniform arrays of GaAs microlenses (MLs) in three shapes (peak, gauss, and hat) by wet-chemical etching. The MLs with epitaxial InAs QDs embedded prove a light extraction efficiency η as high as 26% (i.e., 15 times enhancement concerning the flat sample η = 1.7%) in a broad spectral band 890–970 nm. The MLs enable a ∼200 nm spatial isolation of epitaxial single QDs (suitable for telecom-band epitaxial QDs too) and a high single-photon purity ($g^{\left(2\right)}\left(0\right)$ = 0.02). The hat-shaped MLs combining with the bottom DBR achieve a total single-photon extraction rate at the first lens of $7\times 10^{6}$ cps ($X^{+}$) with a Purcell factor of ∼2 and light extraction efficiency of 26% which can be further improved by replacing the bottom DBR with a gold mirror. The MLs enhance both XX and *X* exciton emissions in some QDs and their cross-correlations prove a cascade emission for further entanglement application. This work paves a step towards a scalable array of QD single-photon sources and the application of QD photon-pair emission for entanglement.

## Figures and Tables

**Table 1:** Detailed fabrication parameters and shape parameters of the three shapes of MLs.

ML Shape	${\mathrm{SiO}}_{2}$ Mask Diameter	Etching Time	H	W	FWHM
peak	1.9 μm	90 min	0.59 μm	0.05 μm	0.59 μm
gauss	2.2 μm	90 min	0.53 μm	0.08 μm	0.61 μm
hat	2.5 μm	90 min	0.68 μm	0.01 μm	1.04 μm

## Data Availability

The data that support the findings of this study are available from the corresponding author upon reasonable request.
